# Effectiveness, safety, and implementation outcomes of a decentralization program to deliver antivenoms in the Western Brazilian Amazonia: The SAVING program

**DOI:** 10.1371/journal.pntd.0014612

**Published:** 2026-08-03

**Authors:** Wuelton Monteiro, Felipe Murta, Thiago Serrão-Pinto, Vinicius Machado, Altair Seabra de Farias, Alícia Cacau dos Santos, Deugles Cardoso, Elder Augusto Guimarães Figueira, Érica Cristina da Silva Chagas, Luana Souza Habibe, Leonardo da Silva Ipuchima, Nilzoney Ferreira de Souza, Taynnah da Silva Lima, Flávio Santos Dourado, Lucia Montebello, Francisco Edilson Ferreira de Lima-Junior, Nicholas R. Casewell, David G. Lalloo, Fernando Almeida-Val, Marcus Lacerda, Joao Ricardo Nickenig Vissoci, Charles J. Gerardo, Fan Hui Wen, Jacqueline Sachett

**Affiliations:** 1 School of Health Sciences, Universidade do Estado do Amazonas, Manaus, Brazil; 2 Department of Teaching and Research, Fundação de Medicina Tropical Dr. Heitor Vieira Dourado, Manaus, Brazil; 3 Department of Environmental Surveillance, Fundação de Vigilância em Saúde do Amazonas Dra. Rosemary Costa Pinto, Manaus, Brazil; 4 Indigenous Health Secretariat, Ministry of Health, Brasília, Brazil; 5 Secretariat for Health and Environmental Surveillance, Ministry of Health, Brasília, Brazil; 6 Centre for Snakebite Research and Interventions, Liverpool School of Tropical Medicine, Pembroke Place, Liverpool, United Kingdom; 7 Department of Tropical Disease Biology, Liverpool School of Tropical Medicine, Pembroke Place, Liverpool, United Kingdom; 8 Medicine School, Federal University of Amazonas, Manaus, Brazil; 9 Global Global Health Institute, Duke University, Durham, North Carolina, United States of America; 10 Department of Emergency Medicine, Duke University, Durham, North Carolina, United States of America; 11 Bioindustrial Center, Butantan Institute, São Paulo, Brazil; Griffith University - GC Campus: Griffith University - Gold Coast Campus, AUSTRALIA

## Abstract

**Background:**

Snakebite envenoming (SBE) is a major cause of morbidity in mortality in low-income and middle-income countries. In Brazil, SBE disproportionately affects Indigenous populations. Limited access to antivenom treatment is a key determinant of poor outcomes in these populations. We evaluated the effectiveness and feasibility of a decentralized model of antivenom delivery in Indigenous primary health settings in the Brazilian Amazon.

**Methods:**

An implementation study was carried out in four Indigenous Health Poles across Amazonas state, Brazil. Effectiveness was assessed using a pre-post design based on national surveillance data, comparing time to antivenom administration, clinical severity, specific clinical manifestations, and case-fatality before and after implementation. Occurrence of early adverse reactions from antivenom treatment was assessed. Qualitative data were collected through semi-structured interviews with Indigenous patients, health-care providers, and health-system managers to assess acceptability, adoption, feasibility, fidelity, and sustainability of the intervention.

**Principal findings:**

Among 125 patients treated after decentralization, the proportion receiving antivenom within 6 hours after bite was significantly higher than under usual care (75.8% vs 40.8%; OR 4·5, 95% CI 2·6–7·9). Proportion of mild envenomations were more frequent after decentralization (50.4% vs 32.7%; OR 2·1, 95% CI 1·2–3·6). Five deaths (4%) were reported in the usual care group and one death (0.8%) was reported in the intervention group (5.3, 95% CI 0.6–45.9). Three (2.4%) patients had mild skin adverse reactions, which were properly managed without need for hospital transfer. A high acceptability was observed among Indigenous users and health providers, combined with good compliance by the providers with the clinical protocol and improved trust in local health services. Health providers and managers at all levels expressed willingness to support sustained antivenom delivery in the study settings and scale up to other Indigenous areas.

**Conclusion:**

Decentralization improved access to antivenom treatment and reduced poor clinical outcomes. Culturally adapted, decentralized care can be safely implemented in remote settings, with important implications for equitable health system strengthening**.**

## Author summary

Snakebite envenoming is a major public health problem in the Amazon region, especially among Indigenous peoples living in remote forest areas. In Brazil, antivenom treatment has traditionally only been available in hospitals located in urban centers, forcing many patients to travel for hours or days before receiving lifesaving care. These delays increase the risk of severe complications, disability, and death. In this study, we evaluated a program that decentralized antivenom treatment to Indigenous primary healthcare units in the western Brazilian Amazon. Health professionals were trained to diagnose and treat snakebites locally, and Indigenous health centers were equipped to safely store and administer antivenoms. We compared outcomes before and after implementation of the program and also interviewed Indigenous patients, healthcare workers, and health managers about their experiences. After decentralization, patients received treatment much faster, and severe complications became less frequent. Deaths were also reduced. Antivenom treatment was shown to be safe in these remote settings, with only a few mild adverse reactions reported. Indigenous communities and healthcare workers strongly supported the program because it reduced the need for long and difficult travel to urban hospitals and allowed care to be delivered closer to patients’ families, languages, and cultural practices. Our findings show that culturally adapted decentralized antivenom care is feasible, safe, and effective in remote Indigenous territories. This strategy can help reduce health inequities and may serve as a model for improving access to emergency care for other neglected diseases in underserved populations.

## 1. Introduction

Snakebite envenoming (SBE) is a high-impact neglected tropical disease affecting 1.8-2.7 million people annually, causing approximately 81,000–138,000 deaths and 400,000 permanent disabilities [1]. It disproportionately affects vulnerable populations in tropical and subtropical regions, with high morbidity and mortality in rural and impoverished settings [1]. In Brazil, around 32,000 snakebites are officially reported each year, although these figures are widely recognized as underestimates, particularly in Indigenous territories where many cases are not captured by surveillance systems [2,3]. Indigenous populations experience a substantially higher burden, with an incidence up to five times higher and case-fatality rates approximately three times greater than in non-Indigenous populations [4]. Beyond mortality, SBE leads to long-term disability, loss of productivity, stigma, trauma, and persistent health inequities [5,6].

In Brazil, antivenom administration has historically been restricted to hospital settings due to concerns about adverse reactions, need for trained personnel, emergency infrastructure, and cold chain requirements for liquid formulations [3]. This centralized model contributes to substantial delays in care in the Amazon, where patients from remote areas may take days to reach health facilities, increasing the risk of severe complications, disability, and death [4,7].

Decentralization of antivenom delivery has been proposed to improve access.4 This strategy involves shifting treatment to adequately prepared primary healthcare facilities closer to where snakebites occur, reducing delays associated with transport, geography, and sociocultural barriers [8]. It may be cost-effective and scalable, particularly in Indigenous territories, where culturally adapted care, use of Indigenous languages, and integration with traditional practices are critical for health-seeking behavior [9–12].

In this study, we evaluated a decentralized model of antivenom delivery in Indigenous primary health services in the western Brazilian Amazon, aiming to assess its effectiveness, safety, and implementation outcomes within Indigenous Health Poles (IHPs- high level primary care units).

## 2. Materials and methods

### 2.1. Ethics statement

The study was approved by Indigenous community leaders, the coordinators of the SIHDs, and the Indigenous Health Councils of the participating territories. Authorization for the research team to enter Indigenous territories was granted by the National Foundation for Indigenous Peoples (FUNAI; 135/AAEP/2024). Ethical approval was obtained from the Brazilian National Research Ethics Commission (CONEP; 85053424.2.0000.0005). Effectiveness and fidelity outcomes were evaluated using anonymized secondary data extracted from the *Sistema de Informação de Agravos de Notificação* (SINAN; Notifiable Diseases Information System) database, Brazilian Notifiable Diseases Information System. These routinely collected surveillance data included records from male and female patients of all ages treated for snakebite envenoming. Access and use of these data were authorized under a Data Use Commitment Agreement, in accordance with Brazilian regulations governing the use of anonymized secondary health data, as approved by CONEP. For the implementation outcomes assessment, which involved semi-structured interviews with aged ≥18 years Indigenous patients, healthcare providers, and health-system managers, written informed consent was obtained from all participants in Portuguese or, when preferred, in Indigenous languages. Confidentiality was ensured through anonymization of transcripts and secure data storage.

### 2.2. Setting and current policies

This Snakebite AntiVenom Immunoglobulins Need to be Guaranteed (SAVING) Program was conducted in the state of Amazonas, western Brazilian Amazon, within Indigenous territories covered by the Indigenous Health Care Subsystem of the Brazilian Unified Health System (SUS) [13]. Brazil has a territorial extension of 8 511 965 km², of which approximately 14% corresponds to Indigenous territories. Most Indigenous territories are located in the Amazon region, which concentrates more than 60% of the Indigenous population nationwide [14]. Indigenous health care is organized through Special Indigenous Health Districts (SIHDs), which deliver primary health care within ethnocultural contexts and coordinate referrals to urban hospitals when higher levels of care are required [13]. Current SBE treatment policy in Brazil stipulates that antivenom treatment should be carried out in public free-of-charge hospitals [3]. Approximately 2,200 hospitals located in urban areas of 2004 municipalities (36% of the 5,568 Brazilian municipalities) provide free-of-charge antivenom treatment in the country [10]. In the current usual care, when a SBE occurs in an indigenous area, the patient receives wound cleaning and analgesics and is transferred to the hospital for antivenom treatment [4].

### 2.3. Study design

**Fig 1:**
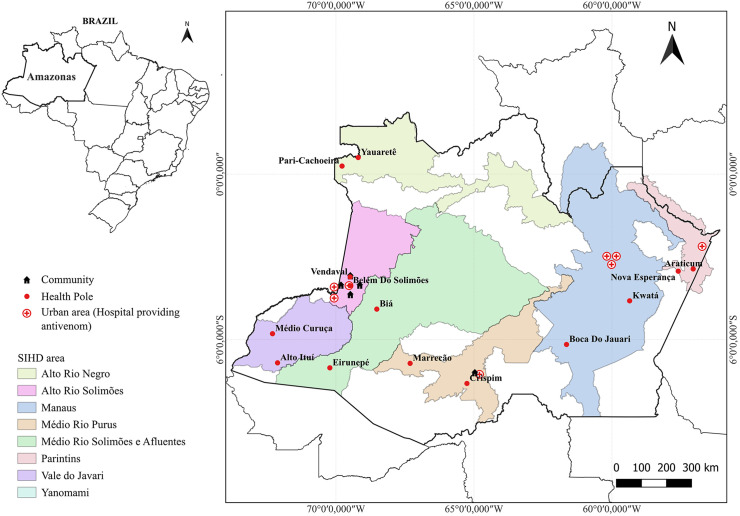
Study area and Indigenous Health Poles included in the SAVING Program. The maps show the geographic distribution of the seven Special Indigenous Health Districts (colored areas) and 14 Indigenous Health Poles (red dots) included in the SAVING Program in Amazonas state, western Brazilian Amazon. The map was produced by the authors using QGIS software and publicly available geographic data from the Brazilian Institute of Geography and Statistics (IBGE; *Instituto Brasileiro de Geografia e Estatística*), freely accessible under the Brazilian Access to Information Law (Law 12,527/2011) [37]. Instituto Brasileiro de Geografia e Estatística. Malha Municipal. 2025 [cited 3 Jan 2026]. Available: https://www.ibge.gov.br/geociencias/organizacao-do-territorio/malhas-territoriais/15774-malhas.html.

**Fig 2:**
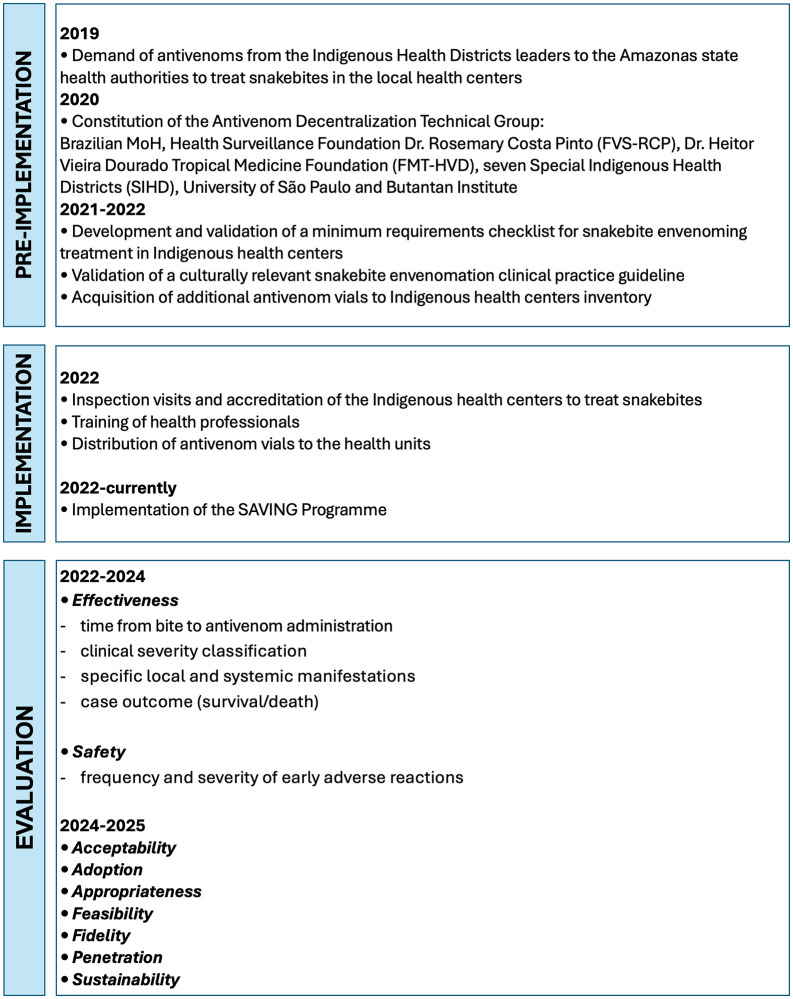
Study design, with operational aspects of the pre-implementation, implementation and evaluation phases of the SAVING Program.

The SAVING Program is an implementation study with an uncontrolled quasi-experimental pre-post design conducted in Indigenous primary health-care facilities in the western Brazilian Amazon. Study design and pre-implementation aspects have been previously described [8]. The intervention was implemented and evaluated in four IHPs located in the Alto Solimões SIHD (Vendaval and Belém do Solimões), the Manaus SIHD (Kwatá), and the Alto Rio Negro SIHD (Yauaretê) (Fig 1). These IHPs were selected based on predefined criteria, including continuous electricity supply to ensure safe storage of liquid antivenoms, availability of full-time physicians authorized to prescribe antivenom, documented SBE cases in the previous three years, and operational capacity to refer severe cases to hospital care when required [15,16]. SAVING Program was based upon Brazilian Ministry of Health SBE treatment standards, adapted to Amazonian Indigenous context [17]. Fig 2 presents pre-implementation, implementation and evaluation phases of the intervention.

### 2.4. Pre-implementation

The pre-implementation phase was initiated in 2019 following formal demands from leaders of SIHDs in the Amazonas state, who advocated for the availability of antivenom treatment within IHPs. From 2020 to 2022, an Antivenom Decentralization Technical Group was constituted to design and coordinate technical, regulatory, and operational aspects of decentralization. A minimum requirements checklist for snakebite envenoming treatment in Indigenous health centers was validated [15,16]. The checklist assessed readiness across human resources, infrastructure, essential equipment, medicines, and emergency preparedness. In parallel, a culturally relevant clinical practice guideline for SBE was validated, ensuring alignment with national standards while incorporating adaptations appropriate to Indigenous territories in the Amazon [17]. To support decentralized delivery, additional snake antivenom vials were provided by the Brazilian Ministry of Health and incorporated into the inventory of the IHPs.

### 2.5. Implementation phase

The implementation phase began in 2022 with inspection visits to IHPs to verify compliance with the minimum requirements and accredit facilities for antivenom administration. Health professionals working at accredited centers underwent structured training focused on diagnosis of SBE, clinical severity classification, antivenom administration, and recognition and management of adverse reactions. Training activities were designed to ensure patient safety while adapting clinical practices to local resource constraints. One to two months following accreditation and training, antivenom vials were distributed to participating IHPs.

### 2.6. Evaluation phase

The SAVING Program was evaluated for effectiveness and safety, represented by patient-centered outcomes, and by seven qualitative implementation outcomes - acceptability, adoption, appropriateness, feasibility, fidelity, penetration, and sustainability [18].

### 2.7. Effectiveness

In Brazil, SBEs are mandatorily reported conditions via SINAN [10]. Effectiveness outcomes were assessed using routinely collected surveillance data from the SINAN-Venomous Animals, reflecting the real-world performance of the intervention under routine programmatic conditions. SINAN data on effectiveness were obtained or both usual care (pre-intervention period, March 2017 to February 2022) and SAVING Program (post-intervention period, March 2022 to August 2025) patients. Effectiveness was measured by the prevalence of i) patients receiving treatment in an adequate time (≤6 hours from bite); ii) severity rate on admission, before the administration of antivenom, iii) rates of specific local and systemic manifestations on admission, iv) rates of specific local and systemic complciations during the health unit stay, and v) case-fatality rate, compared with the estimates obtained for the pre-implementation period. Clinical severity classification of each snakebite type and respective treatment regimens follows the Brazilian Ministry of Health guidelines [17] and are presented in S1 File. The case-fatality rate was based on the outcome recorded in the surveillance system, in which deaths are classified according to their causal relationship with snakebite envenoming, following the standardized case investigation and SINAN reporting procedures. We hypothesized that decentralization would reduce the time from snakebite to antivenom administration, thereby limiting disease progression before treatment and increasing the proportion of patients presenting with mild envenoming at the time of antivenom administration. These outcomes were defined a priori and were constrained to variables systematically collected within the national surveillance system. No additional data were collected specifically for the effectiveness evaluation. Effectiveness was estimated for four Indigenous Health Poles where the program was launched. Eligible patients were male or female individuals of any age, with a clinical-epidemiological diagnosis of a *Bothrops* (pit viper), *Lachesis* (bushmaster) or *Micrurus* (coral snake) envenomation presenting at the Indigenous Health Poles. Exclusion criteria consisted of patients who were bitten by non-venomous snakes, or those with dry bites, i.e., cases with fang marks but no signs of envenomation [19].

Perceptions on effectiveness were also qualitatively assessed from users by in-depth interviews.

### 2.8. Safety

The primary safety outcome was defined as the occurrence of early adverse reactions (EARs) related to type I hypersensitivity, occurring during antivenom infusion or within 24 hours after antivenom administration. EAR severity was classified according to the World Allergy Organization as follows: Grade I, cutaneous manifestations (e.g., pruritus, erythema, urticaria, and angioedema); Grade II, cutaneous manifestations associated with non-life-threatening cardiovascular or respiratory symptoms; Grade III, life-threatening manifestations, including shock, severe arrhythmias, or bronchospasm; Grade IV, cardiac and/or respiratory arrest; and Grade V, death [20]. The information about frequency and severity of early adverse reactions was extracted from medical records in the intervention group. Healthcare teams were trained to recognize and manage EARs according to a standardized protocol. For Grade I reactions, antivenom infusion was temporarily interrupted, the reaction was treated with intravenous antihistamines, and antivenom administration was resumed after symptom resolution until the full prescribed dose had been administered. For Grade II or higher reactions, antivenom infusion was immediately interrupted, patients received intravenous antihistamines and/or intramuscular adrenaline as clinically indicated and were transferred to the nearest referral hospital for further management.

### 2.9. Implementation outcomes

Qualitative data on acceptability, adoption, appropriateness, feasibility, fidelity, penetration, and sustainability were collected from November 2024 to August 2025, through semi-structured in-depth interviews conducted by experienced researchers. Operational definitions, participants and levels of analysis for each outcome are summarized in Table 1. All Indigenous health care users were interviewed in person in Indigenous villages. Interviews with healthcare providers, and local, state-level and national policy makers were conducted either in person or via on-line video calls (Zoom platform). Fidelity was also assessed from SINAN database, by the completeness of the reporting forms, antivenom use rates, and the consistency between the severity of the case and the antivenom dosage.

**Table 1:** Definitions and data sources of the qualitative implementation outcomes within the SAVING Program.

Outcome	Definition	Participants
Acceptability	*Users:* Satisfaction of SBE patients with the care received at the Indigenous Health Pole. *Providers:* Perception of the providers on the complexity of the intervention, willingness, safety and comfort to administer antivenom, and workload.	Users and healthcare providers
Adoption	Willingness and decision to initiate, implement, and maintain decentralized antivenom delivery.	Healthcare providers and local managers, state-level and national policy makers
Appropriateness	Perceived fit, relevance, or compatibility of the intervention for the Indigenous Health Pole setting.	Healthcare providers and local managers, state-level and national policy makers
Feasibility	Extent to which the intervention was successfully carried out within the Indigenous Health Pole setting.	Healthcare providers and local managers, state-level and national policy makers
Fidelity	Adherence to the care package guideline to treat snakebite patients within the Indigenous Health Pole setting.	Healthcare providers
Penetration	Extent to which intervention was integrated into routine service delivery, including proportion of the total cases treated locally.	Healthcare providers and local managers
Sustainability	Perceived long-term viability, institutional support, and capacity for integration into routine health system operations.	Healthcare providers and local managers, state-level and national policy makers

Abbreviations: SBE: snakebite envenomation; SINAN: *Sistema de Informação de Agravos de Notificação* (Nation System of Diseases Reporting).

### 2.10. Participants

Data were collected from participants, clinicians and policy makers locally, regionally and nationally [10]. Locally, participants included Indigenous patients who received antivenom treatment at Indigenous health centers after implementation of the decentralized model, health-care providers (physicians, nurses and nursing technicians) who treated at least one SBE patient after the implementation of the program, and local health-unit managers. At state level participants included representatives from state-level institutions responsible for health surveillance, immunization logistics, and Indigenous health coordination. Nationally, participants included stakeholders from the Ministry of Health responsible for snakebite surveillance, antivenom regulation and distribution, and Indigenous health policy. We purposefully selected participants from each subgroup to ensure representation of different variations within the groups (S2 File). A single refusal of a state-level representative was observed due to unavailability in all the time options attempted.

### 2.11. Data analysis

Data were extracted from the SINAN database by the Amazonas State Health Surveillance Foundation and provided to the investigators as anonymized Microsoft Excel spreadsheets. Data were reviewed for completeness and consistency before statistical analysis. Baseline demographic, epidemiological, and clinical characteristics were summarized using frequencies in the pre-intervention and post-intervention periods. Effectiveness and fidelity outcomes were analyzed using complete-case data for each variable. Categorical variables were summarized as frequencies and compared between the pre-implementation (usual care) and intervention groups using Pearson’s χ² test or Fisher’s exact test, as appropriate, in a quasi-experimental 1:1 design paired by IHP. Univariate logistic regression models were fitted to estimate odds ratios (ORs) and 95% confidence intervals (CIs) for each outcome. To evaluate the association between the intervention and the outcomes of snakebite severity (mild, moderate, and severe) and time to healthcare (<6 h, 6–12 h, 12–24 h, and >24 h), ordinal logistic regression models were also fitted. Odds ratios (ORs) and corresponding 95% confidence intervals (95% CIs) were estimated under the proportional odds assumption. A two-sided p value <0.05 was considered statistically significant.

Qualitative data were analyzed using deductive content analysis, guided by the predefined implementation outcomes proposed by Proctor et al. (acceptability, adoption, appropriateness, feasibility, fidelity, penetration, and sustainability) [18]. Audio-recorded interviews were transcribed verbatim, anonymized, and imported for analysis. Two researchers independently performed line-by-line coding of all transcripts using an a priori coding framework based on the implementation outcomes (T.S.-P. and A.C.dosS.). Emerging subthemes within each implementation outcome were identified through iterative comparison of coded segments. Coding discrepancies were discussed and resolved by consensus with senior investigators (V.M. and F.M.).

## 3. Results

### 3.1. Effectiveness

A total of 125 snakebites cases was reported in the evaluation period (March 2022 to August 2025). The same number of cases treated under the usual care were selected from March 2017 to February 2022, in the pre-intervention period. The baseline characteristics of both groups were mostly similar (Table 2).

**Table 2:** Baseline characteristics of the Indigenous patients treated by the usual care and after SAVING Program implementation.

Variable ^a^	Intervention group (n, %)	Usual care (n, %)
**Gender**	*125 (100.0%)*	*125 (100.0%)*
Male	73 (58.4%)	79 (63.2%)
**Age groups, years**	*125 (100.0%)*	*125 (100.0%)*
0-15	23 (18.4%)	29 (23.2%)
16-45	68 (54.4%)	75 (60.0%)
46-65	28 (22.4%)	16 (12.8%)
>65	6 (4.8%)	5 (4.0%)
**Anatomical site of the bite**	*124 (99.2%)*	*125 (100.0%)*
Lower limbs	107 (86.3%)	104 (83.2%)
Upper limbs	16 (12.9%)	21 (16.8%)
Lower and upper limbs	1 (0.8%)	0 (0.0%)
**Education, years**	*119 (95.2%)*	*89 (71.2%)*
Illiterate	12 (10.1%)	9 (10.1%)
1-4	43 (36.1%)	69 (77.5%)
5-8	30 (25.2%)	10 (11.2%)
>8	34 (28.6%)	1 (1.2%)
**Perpetrating snake**	*124 (99.2%)*	*123 (98.4%)*
*Bothrops atrox* (lancehead)	122 (98.4%)	113 (90.4%)
*Lachesis muta* (bushmaster)	2 (1.6%)	10 (9.6%)

^a^Completeness of the variable is shown in italics.

Effectiveness data are presented in Table 3. Patients in the intervention group were significantly more likely to receive antivenom within 6 hours of the snakebite than those managed under the usual care model (OR 4.5: 95% CI 2.6, 7.9; *P* < 0.001). Ordinal logistic regression showed that participants in the control group had significantly higher odds of presenting later for care compared with participants who received the SAVING intervention (OR 3.6, 95% CI 2.1-6.2; *P* < 0.001). Compared with the intervention group, patients receiving usual care were significantly more likely to present with moderate (OR 2.1, 95% CI 1.1-3.5; *P* = 0.027) or severe envenoming (OR 2.0, 95% CI 1.0-4.3; *P* = 0.059) on admission, before antivenom administration. In an ordinal logistic regression evaluating severity as the outcome, the control group had significantly higher odds of more severe envenomation compared with the SAVING group (OR 2.0, 95% CI 1.2-3.3; *P* = 0.005).

**Table 3:** Effectiveness outcomes in Indigenous patients treated by the usual care and after SAVING Program implementation.

Variable^a^	Intervention group (n, %)	Usual care (n, %)	Odds ratio, 95% confidence interval	*P*
**Time to proper medical care (≤6 hours)**	*124 (99.2%)*	*120 (96.0%)*		
Yes	94 (75.8%)	49 (40.8%)	1	
No	30 (24.2%)	71 (59.2%)	4.5 (2.6, 7.9)	<0.001
**Categories of time to treatment (hours)** ^**b,c**^				
0-6	94 (75.8%)	49 (40.8%)	1	
6-12	11 (8.9%)	37 (30.8%)	5.6 (2.7, 12.3)	<0.001
12-24	11 (8.9%)	16 (13.3%)	2.8 (1.2, 6.9)	0.021
>24	8 (6.4%)	18 (15.0%)	3.9 (1.6, 10.2)	0.004
Ordinal logistic regression	...	...	3.6 (2.1, 6.2)	<0.001
**Case severity** ^**b,c**^	*125 (100.0%)*	*122 (97.6%)*		
Mild	63 (50.4%)	40 (32.7%)	1	
Moderate	47 (37.6%)	62 (50.9%)	2.1 (1.1, 3.5)	0.027
Severe	15 (12.0%)	20 (16.4%)	2.0 (1.0, 4.3)	0.059
Ordinal logistic regression	...	...	2.0 (1.2, 3.3)	0.005
**Local manifestations** ^**b**^	*118 (94.4%)*	*121 (96.8%)*		
Pain	115 (97.5%)	117 (96.7%)	1.3 (0.3, 5.9)	>0.999
Swelling	114 (96.6%)	110 (90.9%)	2.6 (0.8, 8.4)	0.182
Bruising	37 (31.4%)	31 (25.6%)	0.8 (0.4, 1.3)	0.326
Bleeding	1 (0.9%)	8 (6.6%)	8.3 (1.0, 67.3)	0.039
Blisters	1 (0.9%)	0 (0.0%)	…	0.987
**Systemic manifestations** ^**b**^	*118 (94.4%)*	*121 (96.8%)*		
Bleeding	11 (8.8%)	17 (14.1%)	1.6 (0.7, 3.6)	0.257
**Clotting time** ^**b**^	*96 (76.8%)*	*110 (88.0%)*		
Not performed	53 (55.2%)	42 (38.2%)	…	…
Normal	33 (34.4%)	35 (31.9%)	1	
Abnormal	10 (10.4%)	33 (29.9%)	3.1 (1.3, 7.3)	0.008
**Local and systemic complications** ^**d**^				
Blisters	1 (0.9%)	0 (0.0%)	…	0.987
Necrosis	1 (0.9%)	1 (0.83%)	…	>0.999
Secondary infection	1 (0.9%)	11 (9.1%)	11.7 (1.5, 92.1)	0.006
Amputation	1 (0.9%)	0 (0.0%)	…	0.987
Compartment syndrome	0 (0.0%)	1 (0.8%)	…	>0.999
Functional deficit	0 (0.0%)	2 (1.7%)	…	0.511
Acute renal injury	0 (0.0%)	7 (5.8%)	7.3 (0.9, 59.8)	0.016
**Outcome** ^**e**^	*115 (92.0%)*	*113 (90.4%)*		
Discharged	114 (99.1%)	108 (95.6%)	1	
Death	1 (0.9%)	5 (4.4%)	5.3 (0.6, 45.9)	0.204

^a^ Completeness of the variable is shown in italics.

^b^ Data collected on admission, before antivenom administration.

^c^ Univariate logistic regression was used for binary comparisons. Ordinal logistic regression was used to evaluate the association between the intervention and the ordered categories of time to treatment and snakebite severity grading.

^d^ Data collected throughout the entire stay at the health unit.

^e^ Data recorded in the surveillance system, in which deaths are classified according to their causal relationship with snakebite envenoming, following the standardized case investigation and SINAN reporting procedures.

On admission, local bleeding (OR 8.3, 95% CI 1.0-67.3; *P* = 0.039) and non-clotting blood (OR 3.1, 95% CI 1.3-7.3; *P* = 0.008) were significantly more reported in the pre-intervention group. During health unit stay, secondary bacterial infection (OR 11.7, 95% CI 1.5-92.1; *P* = 0.006) and acute kidney injury (OR 7.3, 95% CI 0.9-59.8; *P* = 0.016). Other local and systemic manifestations on admission and complications during follow-up did not differ significantly between groups. Five deaths (4%) were reported in the usual care group and one death (0.8%) was reported in the intervention group (5.3, 95% CI 0.6–45.9). The death recorded in the intervention groups had hemorrhagic stroke as its immediate cause, in a patient who took 48 hours to receive antivenom.

User’s perception of the treatment’s effectiveness was positive, with the most notable mention being rapid pain relief after treatment at the health facility. Patients also considered the time spent at the facility sufficient to improve their health before returning home. Reports suggest a strong influence of negative experiences prior to implementation, especially long hospital stays.

### 3.2. Safety

Premedication with antihistamines to prevent allergic reactions after antivenom treatment was adopted as recommended. Three patients (2.4%) developed Grade I early adverse reactions, all of which resolved after temporary interruption of antivenom infusion and treatment with intravenous antihistamines, allowing completion of the prescribed antivenom regimen. No Grade II–V early adverse reactions (including anaphylactoid reactions) were observed.

### 3.3. Acceptability

The positive users’ acceptability was closely related to the proximity of health-care facilities providing SBE treatment and the convenience of receiving care locally. Key aspects included easier access, communication in Indigenous languages, stronger connections with health staff, and the possibility of joint care involving Indigenous healers and health professionals. These features contrasted with barriers experienced before antivenom decentralization, particularly the need to travel to urban hospitals. Negative responses were mainly related to insufficient information about the care received during IHP stay, lack of immediate rescue or transportation in some situations, and limited dissemination of information about the intervention within villages. From the providers’ perspective, acceptability among users was perceived as high and associated with the avoidance of travel outside Indigenous territories, bilingual assistance, appropriate diet and accommodation, and the involvement of family members and Indigenous healers. Reduction in severe cases, combined with culturally adapted patient care in indigenous health units culminated in greater credibility for the professional team.

Providers also reported high acceptability of the intervention itself. Participation in training activities was high, although initial concerns regarding safety and comfort in administering antivenom outside hospital settings were reported. Providers’ self-confidence increased over time as antivenom was administered without adverse reactions and positive outcomes became evident. Although the intervention increased local workload, this was offset by reductions in the effort, resources, and time required for patient transfers to urban hospitals, alongside improved professional autonomy and job satisfaction.

### 3.4. Adoption

Decentralized antivenom treatment was mentioned as a longstanding and persistent demand from Indigenous Health Poles. Health providers were aware of SBE burden and mostly understood the innovation, demonstrating a willingness to implement the new practice into existing workflows. The SAVING care package guideline was adopted by providers in all health units. Directed training of the nursing staff in nursing procedures was considered a relevant measure for adoption. Motivated and committed champions were identified among the providers, which was understood as decisive to promote the program. Training of newly arrived nurses and physicians by the pioneer staff ensured the adoption of the intervention in some poles.

### 3.5. Appropriateness

Although the program was designed to be provided within a primary health unit, some patients resisted going to the pole or were not authorized by the family or the traditional indigenous healer, and were treated with antivenom in the villages, including in temporary shelters in the forest. To balance rigor and practicality, antivenoms were transported in a refrigerated container, and contact with the physician was maintained by cell phone.

Communication between non-indigenous providers and patients was facilitated by bilingual Indigenous health agents. Providers reported that the available boats do not offer conditions for transporting a snakebite patient in a comfortable position. Snakebite patients needed to stay in the health unit overnight, sometimes for days, and health staff must improvise meals and suitable spaces for accommodation. However, this allowed inpatients to consume food while respecting the dietary prohibitions inherent to indigenous culture. According to Indigenous culture, SBE patients cannot have contact with pregnant or menstruating women or their contacts, or with people who have recently had sexual intercourse. This creates the need for a designated space for accommodation, which is a concern for professionals. Professionals demonstrated tolerance towards traditional practices performed by healers or family members for patients within the health unit.

A barrier identified by managers at all levels is the non-interoperability between the Indigenous Health Care Information System (SIASI), which is the information system used by the Brazilian government to collect and manage data on the health of indigenous populations, and the SINAN database, in which SBEs must be reported. This means and increased workload for health professionals and hinders communication between epidemiological surveillance and the indigenous health system.

### 3.6. Feasibility

Decentralized antivenom delivery was reported as feasible across Indigenous health units following training and accreditation processes, with providers describing confidence in administering antivenom in local settings. Reported challenges included staff turnover, heterogeneity in infrastructure and resources across health units, and logistical constraints related to antivenom storage, transportation, and timely replenishment. However, there were no reports of interruptions in the distribution of antivenoms to health units. Internet availability in the villages is a positive point reported by providers, offering the possibility of contacting specialists during case management. The health units are not able to provide immediate transfer of severe patients to hospitals in some situations, due to the availability of few boats, climate issues and low navigability of the river at certain months of the year. Antivenoms are distributed to indigenous areas using vaccine cold chain, and there was concern about power outages and lack of maintenance of generators and refrigerators in the village.

### 3.7. Fidelity

We observed some flaws in case reporting and inconsistent antivenom dosages during case management. Completeness of the case notification form was very good for most variables. Total completeness was observed for demographics, case severity, antivenom treatment and antivenom dosage. The whole blood clotting test was not performed in 55.2% of patients in the intervention group and 38.2% of those receiving usual care. Additionally, the case outcome (death or survival) was not filled in for 10 cases (8.0%) (Table 4). All cases of the intervention group received antivenom, while 118 (94.4%) of the usual care group were treated with antivenoms. Antivenom dosage was consistent with the patients’ clinical severity in 94 cases (75.2%). Frequency of deviations in antivenom dosage from recommendations was similar in the intervention and usual care groups.

**Table 4:** Fidelity outcomes in Indigenous patients treated by the usual care and after SAVING Program implementation.

Variable^a^	Intervention group (n, %)	Usual care (n, %)	Odds ratio, 95% confidence interval	*P*
**Antivenom treatment** ^**b**^	*125 (100.0%)*	*125 (100.0%)*		
Yes	125 (100.0%)	118 (94.4%)	…	0.014
**Antivenom dosage** ^**c**^	*125 (100.0%)*	*118 (94.4%)*		
As recommended	94 (75.2%)	79 (66.9%)	1	
Underdosage	4 (3.2%)	3 (2.4%)	0.9 (0.2, 4.1)	>0.999
Overdosage	27 (21.6%)	34 (28.8%)	1.5 (0.8, 2.7)	0.176
Not informed dosage	0 (0.0%)	2 (1.7%)	...	...

^a^Completeness of the variable is shown in italics.

^b^All patients included in the analysis were eligible to receive antivenom treatment according to the Brazilian Ministry of Health guidelines.

^c^Clinical severity classification of each snakebite type and respective treatment dosages follows the Brazilian Ministry of Health guidelines and are presented in S1 File.

^d^Data collected throughout the entire stay at the health unit.

^e^Data recorded in the surveillance system, in which deaths are classified according to their causal relationship with snakebite envenoming, following the standardized case investigation and SINAN reporting procedures.

### 3.8. Penetration

Even with dissemination activities carried out by community leaders and health professionals, some users still lacked information about the intervention. Providers suggested that neighboring poles could be notified about the possibility of transporting SBE patients to those providing antivenom, which allows for greater penetration of the intervention. There were several suggestions from providers to extend the intervention to other indigenous areas that could also benefit from the intervention.

### 3.9. Sustainability

Sustainability was perceived as dependent on continued training and supervision, stable staffing, and reliable antivenom supply chains. Concerns were raised regarding long-term funding, workforce turnover, and potential disruptions to antivenom procurement and distribution. Local managers link the receipt of new vials of antivenom into their stocks preventing intervention discontinuity to possessing an effective epidemiological surveillance. Reduction in patient transfers to urban areas represent cost savings and add long-term value to the program. Initiatives such as the *Programa Mais Médicos* (More Doctors Program) helped to mitigate the difficulty in hiring doctors to work in indigenous health units and avoided the discontinuity of the intervention. Managers also expressed concern about the cold chain, since the indigenous health system has few technicians responsible for preventive maintenance and repair of solar panels and refrigerators.

Providers and managers understand that discontinuing the intervention would be a setback in the care process in indigenous health units with a profound negative impact on users trust in the health system.

## 4. Discussion

This study shows that decentralized antivenom delivery within IHPs in the Brazilian Amazon is safe, feasible, acceptable, and associated with clinically meaningful improvements in the timeliness of SBE care. Although antivenom is the mainstay of SBE treatment, misconceptions regarding its safety have historically restricted its use to hospital settings, limiting access for rural and Indigenous populations [10]. Improvements in antivenom quality have reduced these risks, enabling alternative models of care [3].

By enabling antivenom administration closer to where SBE occurs, decentralization reduced treatment delays and was associated with less severe clinical presentations at admission. These findings are consistent with evidence that time to antivenom administration is a key determinant of clinical outcomes [21,22]. In the Amazon, long distances, river-based transport, seasonal variability, and reliance on referral hospitals create structural delays incompatible with optimal management [23,24]. Our findings suggest that decentralization addresses both geographic and health system barriers, aligning with the WHO roadmap recommending expanded access to antivenom [1].

Beyond clinical outcomes, this study demonstrates how decentralized antivenom delivery can be operationalized within complex, real-world health systems. While the effectiveness of antivenom is well established, evidence on scalable delivery models for low-resource and Indigenous settings remains limited. By combining routinely collected surveillance data with qualitative implementation research, this study provides evidence on both intervention effectiveness and the processes that influence successful implementation, thereby informing health system decision-making [25,26].

The study also highlights the balance between fidelity to clinical protocols and adaptation to local contexts. Adherence to treatment standards was high; however, adaptations were required to accommodate variability in infrastructure, staffing, communication, and patient flow, enabling feasibility while maintaining continuity of care. This is consistent with implementation science frameworks that recognize context-sensitive adaptation as essential for effective and equitable implementation [26,27]. This flexibility also underpinned acceptability. Treatment delivered within Indigenous territories was consistently perceived as more acceptable and trustworthy than hospital-based care, reinforcing evidence that culturally safe, community-embedded models are essential for improving access and equity [4,11,28]. Although antivenom was occasionally administered in villages rather than at the designated IHPs, representing a deviation from the planned implementation strategy, this experience also demonstrated that antivenom could be safely delivered in even more decentralized settings than originally anticipated. These findings suggest that future implementation strategies may be further adapted to other culturally appropriate contexts.

Successful implementation was supported by several contextual factors, including early engagement of Indigenous leaders and health authorities, co-design of the intervention with key stakeholders, standardized training of multidisciplinary teams, telehealth support for clinical decision-making, clear clinical protocols, and integration with the Indigenous Health Care System. Together, these components promoted stakeholder buy-in, strengthened implementation fidelity, and supported the safe decentralization of antivenom administration in remote settings.

These findings also have implications beyond the Brazilian Indigenous Health Care System. Although implemented within a unique organizational context, many of the implementation principles identified in this study are likely to be transferable to other remote and underserved settings where delayed access to antivenom remains a major cause of snakebite mortality and disability. While specific operational arrangements should be adapted to local health system structures, the core elements of stakeholder engagement, competency-based training, standardized treatment protocols, telehealth support, and continuous implementation evaluation may provide a useful framework for scaling up decentralized antivenom delivery in other endemic regions.

Scaling up decentralized antivenom delivery will require sustained investment in health system capacity, including reliable cold-chain infrastructure, uninterrupted antivenom supply, ongoing competency-based training, telehealth support, supportive supervision, and routine monitoring of implementation indicators. Although referral of patients to neighboring Indigenous Health Poles with antivenom availability may serve as an interim strategy in selected settings, expanding local treatment capacity remains the preferred approach because it minimizes delays to antivenom administration and reduces the logistical burden associated with inter-facility transport.

The findings of this study informed the development of AJURI, a scalable implementation platform designed to translate lessons from the SAVING Program into operational tools, including readiness requirements, standardized training, clinical protocols, and supply-chain governance. By addressing barriers such as workforce turnover and logistical constraints, AJURI aims to support scale-up while maintaining fidelity and local adaptability [29]. The implementation findings also identify opportunities to further strengthen decentralized antivenom delivery. Community concerns regarding limited awareness of antivenom availability highlight the need for ongoing health education and communication strategies involving Indigenous leaders, community health workers, and local health professionals. In addition, managers emphasized the importance of improving interoperability between Indigenous and national health information systems to strengthen surveillance, facilitate program monitoring, and support evidence-informed decision-making. Addressing these implementation barriers will be essential to support sustainable scale-up of decentralized antivenom delivery and reduce inequities in access to life-saving treatment for remote Indigenous populations.

This study has some limitations. First, the uncontrolled quasi-experimental before-and-after design, which relied on historical controls, cannot completely exclude the influence of secular trends, temporal changes in healthcare delivery, or residual confounding. Second, because this was a pragmatic evaluation embedded within the SAVING Program, effectiveness outcomes relied on routinely collected surveillance data, which is affected by incomplete reporting and limited control over data quality. These characteristics represent an inherent trade-off of evaluating interventions under routine health service conditions. Operational challenges identified during implementation, including variability in adherence to some protocol components, were evaluated as implementation outcomes reflecting fidelity and feasibility rather than solely methodological limitations. Finally, the relatively small number of severe outcomes, particularly complications and deaths, limited the statistical power to detect differences in rare outcomes.

Strengths of this study include its evaluation of a real-world health system intervention to improve access to antivenom for Indigenous populations in the Brazilian Amazon, yielding findings with high programmatic relevance and external validity. The multi-methods design integrated effectiveness and implementation outcomes under routine healthcare conditions. The study also addresses an important evidence gap by focusing on a historically underserved population that has been largely excluded from implementation research on snakebite care.

## 5. Conclusions

Decentralized antivenom delivery within IHPs is an effective, safe, feasible and acceptable strategy that significantly improved access to SBE care in remote settings. Implementation outcomes highlight the importance of cultural adaptation, flexible delivery models, and sustained system-level support. SAVING Program offers a scalable and equitable pathway for strengthening snakebite care and provides a transferable model for addressing other neglected, time-critical conditions in resource-constrained and Indigenous contexts.

## Supporting information

S1 File Clinical grading and antivenom regimens for snakebite treatment according to the Brazilian Ministry of Health. (DOCX)

S2 File Characteristics of the study participants enrolled in the evaluation phase to assess implementation outcomes of the SAVING Program. (DOCX)
