# Supplementary material for: Effectiveness, safety, and implementation outcomes of a decentralization program to deliver antivenoms in the Western Brazilian Amazonia: The SAVING program
Source: PLoS Negl Trop Dis. 2026 Aug 3;20(8):e0014612. doi: 10.1371/journal.pntd.0014612 (PMC13432097; doi:10.1371/journal.pntd.0014612)
Supplement: S1 File — (DOCX) [file pntd.0014612.s001.docx]

**S1 file**

Clinical grading and antivenom regimens for snakebite treatment according to the Brazilian Ministry of Health.

| **Type of snakebite** | **Antivenom** | **Clinical grading** | **Dosage (number of vials)** |
| --- | --- | --- | --- |
| *Bothrops* (pit vipers) | *Bothrops* antivenom (BAV)^a^ or *Bothrops-Lachesis* antivenom (BLAV)^b^ | **Mild**: Mild local manifestations, with pain, swelling and bruising. | 3 |
|  |  | **Moderate**: Pain, evident swelling and bruising, and minor systemic bleeding. | 6 |
|  |  | **Severe**: Intense local manifestations and massive bleeding. | 12 |
| *Lachesis*^c^ (bushmasters) | *Bothrops-Lachesis* antivenom (BLAV)^b^ | **Moderate**: Local manifestations, minor systemic bleeding, without vagal syndrome. | 10 |
|  |  | **Severe**: Intense local manifestations, massive bleeding, and vagal syndrome (abdominal cramps, diarrhea, bradycardia, hypotension). | 20 |
| *Micrurus* (coral snakes) | *Micrurus* antivenom (MAV)^d^ | **Mild**: Mild pain and paresthesia in the bitten limb. | No antivenom required |
|  |  | **Moderate**: Mild pain or paresthesia in the bitten limb, manifestations of decreased muscle strength such as eyelid ptosis, blurred vision, without respiratory paralysis. | 10 |
|  |  | **Severe**: Mild pain or paresthesia in the bitten limb, manifestations of decreased muscle strength such as eyelid ptosis, difficulty getting up and walking, dysphagia and salivation, dyspnea that can progress to respiratory paralysis. | 20 |

^a^ Each vial of BAV contains heterologous horse F(ab’)2, neutralizing at least 50 mg of the reference venom of *Bothrops jararaca* in mice, phenol (35 mg maximum) and physiological solution 0.9% q.s. 10 mL.

^b^ Each vial of BAV contains heterologous horse F(ab’)2, neutralizing at least 50 mg and 30 mg of the reference venoms of *Bothrops jararaca* and *Lachesis muta*, respectively, in mice, phenol (35 mg maximum) and physiological solution 0.9% q.s. 10 mL.

^c^ Due to the potential severity of *Lachesis* bites, they are considered clinically moderate or severe, and there are no mild cases. Diagnosis is mainly clinical and epidemiological, with no routine laboratory test to confirm the type of circulating venom. For areas where there is overlap in the geographical distribution of snakes *Bothrops* and *Lachesis*, such as in the Brazilian Amazon, the differential diagnosis is possible only with the identification of the animal or, in the case of *Lachesis* bites, the possibility of development of vagal manifestations.^1^

^d^ Each vial of MAV contains heterologous horse F(ab’)2, neutralizing at least 15 mg of the reference venom of *Micrurus frontalis* in mice, phenol (35 mg maximum) and physiological solution 0.9% q.s. 10 mL.

**Reference**

1. Brazilian Ministry of Health (2009) Caderno 14-Acidentes por Animais Peçonhentos. Guia de vigilância epidemiológica. Brasília: Brazilian Ministry of Health. p 23.
