# Supplementary material for: Effectiveness, safety, and implementation outcomes of a decentralization program to deliver antivenoms in the Western Brazilian Amazonia: The SAVING program
Source: PLoS Negl Trop Dis. 2026 Aug 3;20(8):e0014612. doi: 10.1371/journal.pntd.0014612 (PMC13432097; doi:10.1371/journal.pntd.0014612)
Supplement: S2 File — (DOCX) [file pntd.0014612.s002.docx]

**S2 File**

Characteristics of the study participants enrolled in the evaluation phase of the SAVING Programme.

| **Variable** | **Intervention group (n, %)** |
| --- | --- |
| Health care users | • 8 participants  • 8 males |
| Health care providers | • 13 participants  • 8 females, 5 males  • 2 of Indigenous ethnicity  • Median age: 35 years  • 9 registered nurses, 2 physicians and 2 nurse assistants  • Work experience: 1-20 years |
| Health system managers | • 18 participants  • Level of care: local level 9; state-level 4; and national level 5 participants  • 10 females, 8 males  • 1 of Indigenous ethnicity  • Median age: 40 years  • 1 BSc, 9 MBA, 4 MSc and 4 with PhD degrees  • Work experience: 3-45 years |
