# Supplementary material for: Effectiveness, safety, and implementation outcomes of a decentralization program to deliver antivenoms in the Western Brazilian Amazonia: The SAVING program
Source: PLoS Negl Trop Dis. 2026 Aug 3;20(8):e0014612. doi: 10.1371/journal.pntd.0014612 (PMC13432097; doi:10.1371/journal.pntd.0014612)
Supplement: Attachment — Submitted filename: Rebuttal letter.docx [file pntd.0014612.s004.docx]

**Dear Editor,**

We sincerely thank you and the reviewers for the careful evaluation of our manuscript and for the constructive and insightful comments provided. We are grateful for the opportunity to revise our work and believe that the suggestions have substantially improved the clarity, rigor, and overall quality of the manuscript. We also appreciate the reviewers' positive comments regarding the relevance and potential impact of our study. Their thoughtful feedback has helped us strengthen both the presentation and interpretation of our findings. In response to the reviewers' recommendations, we have carefully revised the manuscript and addressed each comment point by point. All changes made in the revised manuscript are highlighted, and detailed responses are provided below. We hope that the revisions satisfactorily address all concerns.

**Methods**

Reviewer #1:

It would be helpful to confirm whether the outcome measures were pre-specified. If they were pre-specified, is this available on a protocol that is/can be available via a repository or database.
**Response:** We thank the reviewer for this important comment. This study was designed as a real-world implementation evaluation using an uncontrolled quasi-experimental before-and-after design. The effectiveness outcomes were defined a priori and were constrained to variables systematically collected within the Brazilian National Notifiable Diseases Information System (SINAN), including ***i) time to antivenom administration, ii) severity rate at time of administration of antivenom, iii) rates of specific local and systemic manifestations, and iv) case-fatality rate***, compared with the estimates obtained for the pre-implementation period. These outcomes were selected before the analyses because they represent the standardized indicators systematically collected through the national surveillance system and were considered the most appropriate measures to evaluate the real-world impact of the decentralization program. The study was not prospectively registered, but it was conducted according to the study plan approved by the relevant institutional and research ethics committees before implementation. ***To clarify this point, we have revised the Methods section.***

Under safety subheading in methods, what was the definition for 'early adverse reactions' that was used in this study?

**Response**: The primary safety outcome was defined as the occurrence of early adverse reactions (EARs) related to type I hypersensitivity, occurring during antivenom infusion or within 24 hours after antivenom administration. EAR severity was classified according to the World Allergy Organization as follows: Grade I, cutaneous manifestations (e.g., pruritus, erythema, urticaria, and angioedema); Grade II, cutaneous manifestations associated with non-life-threatening cardiovascular or respiratory symptoms; Grade III, life-threatening manifestations, including shock, severe arrhythmias, or bronchospasm; Grade IV, cardiac and/or respiratory arrest; and Grade V, death [1]. The information about frequency and severity of early adverse reactions was extracted from medical records in the intervention group. Healthcare teams were trained to recognize and manage EARs according to a standardized protocol. For Grade I reactions, antivenom infusion was temporarily interrupted, the reaction was treated with intravenous antihistamines, and antivenom administration was resumed after symptom resolution until the full prescribed dose had been administered. For Grade II or higher reactions, antivenom infusion was immediately interrupted, patients received intravenous antihistamines and/or intramuscular adrenaline as clinically indicated and were transferred to the nearest referral hospital for further management.

Reference:

1. World Allergy Organization. Perioperative Allergic Reactions [Internet]. 2019 [cited 2019 Jun 27]. Available from: https://www.worldallergy.org/education- and-programs/education/allergic-disease-resource-center/professionals/allergy- to-anesthetic-agents.

A statistical analysis plan should be added.

**Response:** We thank the reviewer for this valuable suggestion. We agree that a more detailed description of the statistical analysis would improve the clarity and reproducibility of the study. We have expanded the Statistical Analysis section to provide additional details on the analytical approach, including the descriptive analyses, statistical tests used to compare the pre-implementation and intervention groups, estimation of odds ratios and 95% confidence intervals, handling of missing data through complete-case analyses, and the statistical significance threshold. Because this was a real-world implementation study using routinely collected surveillance data, no additional analyses beyond those specified in the Methods were performed.

Was a power calculation performed to guide the sample size?

**Response**: Thank you for this comment. An a priori sample size calculation was not performed because this was a pragmatic implementation study that included all consecutive snakebite cases treated during the predefined evaluation period (March 2022 to August 2025), with an equal number of consecutive cases selected from the pre-intervention period for comparison. To address the reviewer's concern, we performed a retrospective sample size justification based on the primary outcome of delayed access to antivenom (time from snakebite to antivenom administration >6 hours). The observed proportion of delayed treatment decreased from 59.2% in the pre-intervention period to 24.2% in the post-intervention period. Assuming a two-sided significance level of 0.05, 80% power, independent observations, and equal allocation between groups, approximately 62 participants per group would be required to detect this observed difference (124 in total). Our study included 125 participants in each group (250 in total), indicating that the available sample was adequate for evaluating this outcome.

Outcome measures should be more clearly defined (eg describe in paper what is 'severity rate', and until what time after antivenom was mortality recorded).

**Response:** We thank the reviewer for this valuable comment. We agree that the effectiveness outcomes should be more clearly defined. We have revised the Methods section accordingly. Specifically, we now clarify that clinical severity was classified according to the Brazilian Ministry of Health criteria for each type of snake envenoming, as detailed in Supplementary File 1 (Clinical grading and antivenom regimens for snakebite treatment according to the Brazilian Ministry of Health). This reference was inadvertently omitted from the original text.

We also clarified the definition of case-fatality. Case outcome (death or survival) is a mandatory field in the Brazilian National Notifiable Diseases Information System (SINAN) and is required for case closure. Therefore, the case-fatality rate was based on the final outcome recorded in the surveillance system. No predefined follow-up period was applied; rather, deaths were classified according to the causal relationship between the snakebite envenoming and the fatal outcome, following the standard surveillance procedures adopted by SINAN.

Reviewer #2: A very interesting comparative analysis with a useful outcome. The design, population, statistical analysis are appropriate. Following need to be addressed.
How was the culprit snake identified? [As dosage of antivenom differs with the species]
How was envenomation ascertained? [clotting times not performed in >55% of the intervention group and CT normal in 34.4%. Only 10.4% had abnormal CTs].

**Response:** We thank the reviewer for this important comment. Snakebite envenoming was diagnosed according to the Brazilian Ministry of Health guidelines using a clinical-epidemiological approach based on the history of snakebite together with compatible local and systemic clinical manifestations. The diagnosis of envenoming, as well as severity classification and the indication for antivenom treatment, was based on clinical findings rather than clotting time. In our protocol, clotting time was primarily used to monitor the recovery of coagulation abnormalities following antivenom administration, rather than to establish the diagnosis of envenoming.

The diagnostic criteria for each type of snake envenoming, as well as the severity classification and corresponding antivenom regimens, are detailed in Supplementary File S1 (Clinical grading and antivenom regimens for snakebite treatment according to the Brazilian Ministry of Health).

We agree that clotting time was not performed in a substantial proportion of patients in the intervention group. This was recognized in our study as an important fidelity issue reflecting real-world operational challenges in remote Indigenous health facilities. Nevertheless, among patients in whom clotting time was assessed, the proportion with abnormal results was comparable to that reported in our previous study conducted at a tertiary referral hospital in the Brazilian Amazon [1].

Reference:

1. Oliveira S, C Alves E, S Santos A, F Nascimento E, T Pereira JP, M Silva I, A G Sachett J, S Sarraff LK, Freitas-de-Sousa LA, Colombini M, O Marques H, V G Lacerda M, Sartim MA, Moura-da-Silva AM, L Ferreira LC, S Sano-Martins I, M Monteiro W. Bleeding Disorders in *Bothrops atrox* Envenomations in the Brazilian Amazon: Participation of Hemostatic Factors and the Impact of Tissue Factor. Toxins (Basel). 2020 Aug 29;12(9):554. doi: 10.3390/toxins12090554.

Reviewer #3: Yes, the objectives are clearly stated with a quasi-experimental pre-post design to measure treatment outcomes and qualitative interviews to measure aspects such as acceptability, fidelity, feasibility, etc. While the findings suggest a beneficial effect, confidence intervals indicate some uncertainty regarding the magnitude (as acknowledged in the limitations). The analysis was appropriate. Ethics processes were described in detail and met requirements as reported.

**Response:** We sincerely thank the reviewer for the careful assessment of our manuscript and for the positive comments regarding the study objectives, design, analytical approach, ethical conduct, and interpretation of the findings. We appreciate the recognition of both the strengths of the study and the appropriate acknowledgment of its limitations.

**Results**

Reviewer #1: Line 259 in results the severity of envenoming is presented being a 'response' to the intervention, but this was recorded before the antivenom was administered. I would regard response to treatment as being at a certain time after antivenom - at least 6 hours for something like clotting and longer for other outcome measures.

**Response:** We thank the reviewer for this important observation. Our intention was to show that earlier access to antivenom, achieved through decentralization, resulted in patients presenting for treatment at an earlier stage of envenoming, before progression to more severe clinical manifestations. The sentence has been revised accordingly.

the above comment also applies to table 3. Case severity should be in table 2 (baseline characteristics) rather than being presented as an effectiveness outcome. My interpretation is that the intervention arm had milder cases at enrolment (which may be due to people presenting earlier).

**Response:** We thank the reviewer for this comment. We agree that clinical severity at the time of antivenom administration is not a direct therapeutic response to antivenom itself. However, we respectfully consider that it should remain an effectiveness outcome rather than a baseline characteristic. The primary justification of the SAVING Program was to reduce the time from snakebite to antivenom administration. Clinical severity was assessed immediately before antivenom infusion and therefore reflects disease progression during the interval between the bite and treatment. Earlier access to antivenom is expected to interrupt this progression, resulting in a greater proportion of patients presenting with mild envenoming and fewer progressing to moderate or severe envenoming by the time treatment is initiated. To avoid any misunderstanding, we have revised the manuscript to clarify this rationale.

We added this sentence to the section 2.7 Effectiveness: *“We hypothesized that decentralization would reduce the time from snakebite to antivenom administration, thereby limiting disease progression before treatment and increasing the proportion of patients presenting with mild envenoming at the time of antivenom administration”.*

Likewise for the other outcomes (20WBCT, clinical manifestations of local and systemic evenoming), it is important to state when these were recorded? Were these before antivenom or after?

**Response:** We thank the reviewer for this helpful comment. We agree that the timing of outcome assessment should be clearly specified. We have revised Table 3 and the Methods section to clarify when each outcome was recorded. Clinical severity, clotting time, and local and systemic manifestations were assessed on admission, before antivenom administration, reflecting the patient's clinical status at presentation. In contrast, local and systemic complications were recorded throughout the patient's stay at the health unit, whereas mortality was assessed until case closure according to the Brazilian National Notifiable Diseases Information System (SINAN).

Under safety, report the rate of anaphylactoid reactions (even if this was 0 in both arms). This is a key concern people will have in community administration of antivenom.

**Response:** We thank the reviewer for this important suggestion. We agree that the occurrence of anaphylactoid reactions is a key safety concern when decentralizing antivenom administration to remote health facilities. We have revised the Methods section to explicitly define the primary safety outcome and the World Allergy Organization grading system used to classify early adverse reactions. We have also expanded the Results section to report the frequency and severity of early adverse reactions, including the absence of Grade II–V reactions, demonstrating that no severe hypersensitivity reactions occurred during implementation of the SAVING Program.

Under fidelity subheading, only 43 cases had a 20WBCT - this number differs from the table.

**Response:** We thank the reviewer for identifying this inconsistency. The manuscript has been revised to ensure consistency between the Results text and Table 3.

Under subheading Penetration, there is minor grammatical error first sentence.

**Response:** We thank the reviewer for identifying this grammatical error. The sentence has been revised accordingly.

Table 3 - the numbers and percentages for time to medical care add up to >100%.

**Response:** We thank the reviewer for identifying this source of confusion. The percentages were correct but represented two different categorizations of the same variable (≤6 vs. >6 hours, and the detailed distribution across time intervals). To avoid misinterpretation, Table 3 has been reorganized to clearly distinguish these analyses and improve readability.

Table 3 - presenting individual statistical tests comparing differences in categorical data with 3 group (eg mild, moderate severe) is confusing and doesn't account for these data ordered categorical data. Consider using an ordinal logistic regression to present a single OR, CI and p value to see if there was a tendency toward more or less severe disease. That said, any data moved to baseline charcateristics table would not normally undergo statistical testing.

**Response:** Thank you for this helpful suggestion. Accordingly, we added ordinal logistic regression analyses to evaluate the overall effect of the intervention. Ordinal logistic regression showed that participants in the control group had significantly higher odds of presenting later for care compared with participants who received the SAVING intervention (OR 3.6, 95% CI 2.1-6.2; *P*< 0.001). Moreover, in an ordinal logistic regression evaluating severity as the outcome, the control group had significantly higher odds of more severe envenomation compared with the SAVING group (OR 2.0, 95% CI 1.2-3.3; *P*=0.005). These additional analyses have been incorporated into the Results section to complement the descriptive findings presented in Table 3. These new analyses confirm that the SAVING Program was associated with bot earlier presentation to care and lower snakebite severity.

Reviewer #2: Yes to all, if however, the queries above are addressed sufficiently.

**Response:** We thank the reviewer for the positive assessment of our manuscript and for the constructive comments.

Reviewer #3: Analysis does match the plan and results are clear and detailed. Results are clear and complete- though it would be helpful to have some idea of how many participants of each type participated in the IDIs, to allow for transparency in how different stakeholders may have shaped results. The figures should be reshared as higher quality images.

**Response:** We thank the reviewer for this helpful suggestion. To improve transparency regarding the qualitative component, we have added S2 File (Characteristics of the study participants enrolled in the evaluation phase of the SAVING Programme), which summarizes the characteristics and stakeholder categories of all participants included in the in-depth interviews and focus group discussions. We have also cited this supplementary file in the Methods section.

**Conclusions**

Reviewer #1: Good

Response: We thank the reviewer for the positive assessment of our conclusions.

Reviewer #2: The conclusions are supported by the data presented.
Limitations have not been clearly and adequately described.

**Response:** We thank the reviewer for the positive assessment of our conclusions and for highlighting the need to better describe the study limitations. In response, we have substantially expanded the Limitations section.

Reviewer #3: The conclusions are supported by the data. Limitations are not described in detail, perhaps this can be expanded on. The topic is highly relevant, with positive potential for other settings and countries, expanding on the transferability and resource requirements of this intervention in the discussion, could strengthen the manuscript.

**Response:** We thank the reviewer for the positive assessment of our conclusions and for highlighting the need to better describe the study limitations. In response, we have substantially expanded the Limitations section.

**Editorial and Data Presentation Modifications?**

Reviewer #1: Clearly presented

**Response:** We thank the reviewer for the positive assessment.

Reviewer #2: Minor Revision as mentioned in the previous comments

**Response:** We thank the reviewer for the constructive comments and recommendation. We have carefully addressed all of the points raised and revised the manuscript accordingly.

Reviewer #3: (No Response)

**Response:** n/a.

**Summary and General Comments**

Reviewer #1: Excellent and interting paper. Unique population that are hard to reach and novel approach taken that seems acceptable and appropriate. This papers also marks a step change in terms of thinking about community administration of antivenom, which has traditionally been limited to hospitals primaruly due to concerns of anaphylaxctoid reactions. I would suggest this paper is accepted with the abover modifications.

**Response:** We sincerely thank the reviewer for the thoughtful and encouraging assessment of our manuscript. We have carefully addressed all of the reviewer's comments and revised the manuscript accordingly.

Reviewer #2: Overall a useful study which can impact on public health and contribute to forming policy.

**Response:** We sincerely thank the reviewer for the positive assessment of our study and for recognizing its potential contribution to public health and health policy.

Reviewer #3: This is a well-conducted and detailed evaluation of a highly relevant intervention addressing a recognized barrier to timely snakebite treatment among a vulnerable population group. The mixed-methods approach is a particular strength which allows for assessment of effectiveness as well as factors such as acceptability, fidelity and feasibility.

**Response:** We sincerely thank the reviewer for the thoughtful and encouraging assessment of our manuscript.

Some minor feedback to improve on the manuscript includes:

- Given that a deductive thematic analysis approach was used, the manuscript would benefit from some additional detail regarding the analytic framework that was used, how it was selected and how this informed coding and themes.

**Response**: We thank the reviewer for this valuable suggestion. We agree that the analytic approach required further clarification. We have expanded the Methods section to describe the implementation framework that guided the deductive thematic analysis.

- I was interested to know whether observations were independent or if any clustering by health pole was assessed? It would be relevant to adoption in broader contexts, to understand if the results were uniform or if there were any factors that affected the outcomes across the different study sites.

**Response:** We thank the reviewer for this insightful comment. We agree that exploring variation across Indigenous Health Poles would be valuable to understand implementation in different settings. However, this evaluation included only four participating Indigenous Health Poles, with relatively small numbers of patients treated at each site, limiting the feasibility and statistical power of cluster-level analyses. Importantly, the implementation challenges and qualitative findings were highly consistent across the four poles, suggesting that the observed results were not driven by a single site. We agree that assessing site-level heterogeneity will become increasingly important as the SAVING Program expands to additional Indigenous Health Poles.

- In terms of user acceptability, negative responses were attributed to lack of information regarding care and lack of dissemination in villages. Similarly, managers pointed to the interoperability between health systems databases. Recommendations related to these findings could strengthen the discussion.

**Response:** We thank the reviewer for this valuable suggestion. We agree that the discussion would benefit from translating these implementation findings into practical recommendations. Accordingly, we have expanded the Discussion to highlight potential strategies to address these topics.

- Considering the positive results and the very crucial step this study makes towards addressing the inequitable burden of snakebite, perhaps the discussion can expand on which contextual factors (e.g. training, buy-in, systems support) were essential to the success of the program in the discussion. The authors may want to expand on transferability of findings to other remote/underserved settings internationally, where delayed antivenom access remains a barrier.

**Response:** We thank the reviewer for this insightful suggestion. We agree that discussing the contextual factors underpinning successful implementation and the potential transferability of the findings would strengthen the manuscript. Accordingly, we have expanded the Discussion to highlight the key implementation components that contributed to the success of the SAVING Program.

- The discussion could also be strengthened by further consideration of the resources required to sustain decentralized antivenom administration and implications for scale-up in other communities. This may include discussion of previously mentioned cold-chain availability, for instance. As providers suggested neighboring poles could be notified about the possibility of transporting patients to those providing antivenom, could this be considered an option or would this strain resources and add to healthcare worker burden in those facilities?

**Response:** We thank the reviewer for this insightful suggestion. We agree that the discussion would benefit from further consideration of the resources required to sustain decentralized antivenom administration and its implications for future scale-up. Accordingly, we have expanded the Discussion to address key requirements for sustainability.

- The authors should expand on the strengths and limitations, if word count permits, especially since the use of routine surveillance data was listed as both a strength and a limitation.

**Response:** We thank the reviewer for this helpful suggestion. Accordingly, we have expanded both sections to provide a more balanced discussion of the study's methodological trade-offs.
