# Supplementary material for: Effectiveness, safety, and implementation outcomes of a decentralization program to deliver antivenoms in the Western Brazilian Amazonia: The SAVING program
Source: PLoS Negl Trop Dis. 2026 Aug 3;20(8):e0014612. doi: 10.1371/journal.pntd.0014612 (PMC13432097; doi:10.1371/journal.pntd.0014612)
Supplement: Peer review history

# Peer review: Decision Letter 0

**DOI:** 10.1371/journal.pntd.0014612.r001

**Authors:** José Gutiérrez, Kalana Maduwage

26 Jun 2026

PNTD-D-26-01001

Effectiveness and implementation outcomes of a decentralization program to deliver antivenoms in the Western Brazilian Amazonia: the SAVING Program

PLOS Neglected Tropical Diseases

Dear Dr. Monteiro,

Thank you for submitting your manuscript to PLOS Neglected Tropical Diseases. After careful consideration, we feel that it has merit but does not fully meet PLOS Neglected Tropical Diseases's publication criteria as it currently stands. Therefore, we invite you to submit a revised version of the manuscript that addresses the points raised during the review process.

Please submit your revised manuscript by Jul 26 2026 11:59PM. If you will need more time than this to complete your revisions, please reply to this message or contact the journal office at plosntds@plos.org. When you're ready to submit your revision, log on to https://www.editorialmanager.com/pntd/ and select the 'Submissions Needing Revision' folder to locate your manuscript file.

Please include the following items when submitting your revised manuscript:

* A letter that responds to each point raised by the editor and reviewer(s). You should upload this letter as a separate file labeled 'Response to Reviewers'. This file does not need to include responses to any formatting updates and technical items listed in the 'Journal Requirements' section below.

* A marked-up copy of your manuscript that highlights changes made to the original version. You should upload this as a separate file labeled 'Revised Manuscript with Track Changes'.

* An unmarked version of your revised paper without tracked changes. You should upload this as a separate file labeled 'Manuscript'.

If you would like to make changes to your financial disclosure, competing interests statement, or data availability statement, please make these updates within the submission form at the time of resubmission. Guidelines for resubmitting your figure files are available below the reviewer comments at the end of this letter.

As the corresponding author, your ORCID iD is verified in the submission system and will appear in the published article. PLOS supports the use of ORCID, and we encourage all coauthors to register for an ORCID iD and use it as well. Please encourage your coauthors to verify their ORCID iD within the submission system before final acceptance, as unverified ORCID iDs will not appear in the published article. Only the individual author can complete the verification step; PLOS staff cannot verify ORCID iDs on behalf of authors.

We look forward to receiving your revised manuscript.

Kind regards,

Kalana Maduwage, MBBS, MPhil, PhD, FHEA, FRCP (UK), FRCP (Edin)

Academic Editor

PLOS Neglected Tropical Diseases

José María Gutiérrez

Section Editor

PLOS Neglected Tropical Diseases

Shaden Kamhawi

co-Editor-in-Chief

PLOS Neglected Tropical Diseases

orcid.org/0000-0003-4304-636XX

Paul Brindley

co-Editor-in-Chief

PLOS Neglected Tropical Diseases

orcid.org/0000-0003-1765-0002

**Additional Editor Comments (if provided):**

**Journal Requirements:**

If the reviewer comments include a recommendation to cite specific previously published works, please review and evaluate these publications to determine whether they are relevant and should be cited. There is no requirement to cite these works unless the editor has indicated otherwise.

1) Please upload all main figures as separate Figure files in .tif or .eps format. For more information about how to convert and format your figure files please see our guidelines:

https://journals.plos.org/plosntds/s/figures

2) Please amend your detailed Financial Disclosure statement. This is published with the article. It must therefore be completed in full sentences and contain the exact wording you wish to be published.

i) State what role the funders took in the study. If the funders had no role in your study, please state: "The funders had no role in study design, data collection and analysis, decision to publish, or preparation of the manuscript."

ii) If any authors received a salary from any of your funders, please state which authors and which funders..

If you did not receive any funding for this study, please simply state: u201cThe authors received no specific funding for this work.u201d

3)We have amended your Competing Interest statement to comply with journal style. We kindly ask that you double check the statement and let us know if anything is incorrect.

**Reviewers' comments:**

Reviewer's Responses to Questions

**Key Review Criteria Required for Acceptance?**

As you describe the new analyses required for acceptance, please consider the following:

**Methods**

-Are the objectives of the study clearly articulated with a clear testable hypothesis stated?

-Is the study design appropriate to address the stated objectives?

-Is the population clearly described and appropriate for the hypothesis being tested?

-Is the sample size sufficient to ensure adequate power to address the hypothesis being tested?

-Were correct statistical analysis used to support conclusions?

-Are there concerns about ethical or regulatory requirements being met?

Reviewer #1: It would be helpful to confirm whether the outcome measures were pre-specified. If they were pre-specified, is this available on a protocol that is/can be available via a repository or database.

Under safety subheading in methods, what was the definition for 'early adverse reactions' that was used in this study?

A statistical analysis plan should be added.

Was a power calculation performed to guide the sample size?

Outcome measures should be more clearly defined (eg describe in paper what is 'severity rate', and until what time after antivenom was mortality recorded)

Reviewer #2: A very interesting comparative analysis with a useful outcome. The design, population, statistical analysis are appropriate. Following need to be addressed.

How was the culprit snake identified? [As dosage of antivenom differs with the species]

How was envenomation ascertained? [clotting times not performed in >55% of the intervention group and CT normal in 34.4%. Only 10.4% had abnormal CTs]

Reviewer #3: Yes, the objectives are clearly stated with a quasi-experimental pre-post design to measure treatment outcomes and qualitative interviews to measure aspects such as acceptability, fidelity, feasibility, etc. While the findings suggest a beneficial effect, confidence intervals indicate some uncertainty regarding the magnitude (as acknowledged in the limitations). The analysis was appropriate. Ethics processes were described in detail and met requirements as reported.

**********

**Results**

-Does the analysis presented match the analysis plan?

-Are the results clearly and completely presented?

-Are the figures (Tables, Images) of sufficient quality for clarity?

Reviewer #1: Line 259 in results the severity of envenoming is presented being a 'response' to the intervention, but this was recorded before the antivenom was administered. I would regard response to treatment as being at a certain time after antivenom - at least 6 hours for something like clotting and longer for other outcome measures.

the above comment also applies to table 3. Case severity should be in table 2 (baseline characteristics) rather than being presented as an effectiveness outcome. My interpretation is that the intervention arm had milder cases at enrolment (which may be due to people presenting earlier).

Likewise for the other outcomes (20WBCT, clinical manifestations of local and systemic evenoming), it is important to state when these were recorded? Were these before antivenom or after?

Under safety, report the rate of anaphylactoid reactions (even if this was 0 in both arms). This is a key concern people will have in community administration of antivenom.

Under fidelity subheading, only 43 cases had a 20WBCT - this number differs from the table.

Under subheading Penetration, there is minor grammatical error first sentence.

Table 3 - the numbers and percentages for time to medical care add up to >100%

Table 3 - presenting individual statistical tests comparing differences in categorical data with 3 group (eg mild, moderate severe) is confusing and doesn't account for these data ordered categorical data. Consider using an ordinal logistic regression to present a single OR, CI and p value to see if there was a tendency toward more or less severe disease. That said, any data moived to baseline charcateristics table would not normally undergo statistical testing.

Reviewer #2: Yes to all, if however, the queries above are addressed sufficiently.

Reviewer #3: Analysis does match the plan and results are clear and detailed. Results are clear and complete- though it would be helpful to have some idea of how many participants of each type participated in the IDIs, to allow for transparency in how different stakeholders may have shaped results. The figures should be reshared as higher quality images.

**********

**Conclusions**

-Are the conclusions supported by the data presented?

-Are the limitations of analysis clearly described?

-Do the authors discuss how these data can be helpful to advance our understanding of the topic under study?

-Is public health relevance addressed?

Reviewer #1: Good

Reviewer #2: The conclusions are supported by the data presented.

Limitations have not been clearly and adequately described.

Reviewer #3: The conclusions are supported by the data. Limitations are not described in detail, perhaps this can be expanded on. The topic is highly relevant, with positive potential for other settings and countries, expanding on the transferability and resource requirements of this intervention in the discussion, could strengthen the manuscript.

**********

**Editorial and Data Presentation Modifications?**

Use this section for editorial suggestions as well as relatively minor modifications of existing data that would enhance clarity. If the only modifications needed are minor and/or editorial, you may wish to recommend “Minor Revision” or “Accept”.

Reviewer #1: Clearly presented

Reviewer #2: Minor Revision as mentioned in the previous comments

Reviewer #3: (No Response)

**********

**Summary and General Comments**

Use this section to provide overall comments, discuss strengths/weaknesses of the study, novelty, significance, general execution and scholarship. You may also include additional comments for the author, including concerns about dual publication, research ethics, or publication ethics. If requesting major revision, please articulate the new experiments that are needed.

Reviewer #1: Excellent and interting paper. Unique population that are hard to reach and novel approach taken that seems acceptable and appropriate. This papers also marks a step change in terms of thinking about community administration of antivenom, which has traditionally been limited to hospitals primaruly due to concerns of anaphylaxctoid reactions. I would suggest this paper is accepted with the abover modifications

Reviewer #2: Overall a useful study which can impact on public health and contribute to forming policy.

Reviewer #3: This is a well-conducted and detailed evaluation of a highly relevant intervention addressing a recognized barrier to timely snakebite treatment among a vulnerable population group. The mixed-methods approach is a particular strength which allows for assessment of effectiveness as well as factors such as acceptability, fidelity and feasibility.

Some minor feedback to improve on the manuscript includes:

- Given that a deductive thematic analysis approach was used, the manuscript would benefit from some additional detail regarding the analytic framework that was used, how it was selected and how this informed coding and themes.

- I was interested to know whether observations were independent or if any clustering by health pole was assessed? It would be relevant to adoption in broader contexts, to understand if the results were uniform or if there were any factors that affected the outcomes across the different study sites.

- In terms of user acceptability, negative responses were attributed to lack of information regarding care and lack of dissemination in villages. Similarly, managers pointed to the interoperability between health systems databases. Recommendations related to these findings could strengthen the discussion

- Considering the positive results and the very crucial step this study makes towards addressing the inequitable burden of snakebite, perhaps the discussion can expand on which contextual factors (e.g. training, buy-in, systems support) were essential to the success of the program in the discussion. The authors may want to expand on transferability of findings to other remote/underserved settings internationally, where delayed antivenom access remains a barrier.

- The discussion could also be strengthened by further consideration of the resources required to sustain decentralized antivenom administration and implications for scale-up in other communities. This may include discussion of previously mentioned cold-chain availability, for instance. As providers suggested neighboring poles could be notified about the possibility of transporting patients to those providing antivenom, could this be considered an option or would this strain resources and add to healthcare worker burden in those facilities?

- The authors should expand on the strengths and limitations, if word count permits, especially since the use of routine surveillance data was listed as both a strength and a limitation.

**********

PLOS authors have the option to publish the peer review history of their article (what does this mean?). If published, this will include your full peer review and any attached files.

If you choose “no”, your identity will remain anonymous but your review may still be made public.

**Do you want your identity to be public for this peer review?**  For information about this choice, including consent withdrawal, please see our Privacy Policy.

Reviewer #1: **Yes:** Michael Abouyannis

Reviewer #2: No

Reviewer #3: **Yes:** Inika Sharma

[NOTE: If reviewer comments were submitted as an attachment file, they will be attached to this email and accessible via the submission site. Please log into your account, locate the manuscript record, and check for the action link "View Attachments". If this link does not appear, there are no attachment files.]

**Figure resubmission:**

While revising your submission, we strongly recommend that you use PLOS’s NAAS tool (https://ngplosjournals.pagemajik.ai/artanalysis) to test your figure files. NAAS can convert your figure files to the TIFF file type and meet basic requirements (such as print size, resolution), or provide you with a report on issues that do not meet our requirements and that NAAS cannot fix.

After uploading your figures to PLOS’s NAAS tool - https://ngplosjournals.pagemajik.ai/artanalysis, NAAS will process the files provided and display the results in the "Uploaded Files" section of the page as the processing is complete. If the uploaded figures meet our requirements (or NAAS is able to fix the files to meet our requirements), the figure will be marked as "fixed" above. If NAAS is unable to fix the files, a red "failed" label will appear above. When NAAS has confirmed that the figure files meet our requirements, please download the file via the download option, and include these NAAS processed figure files when submitting your revised manuscript.

**Reproducibility:**

To enhance the reproducibility of your results, we recommend that authors of applicable studies deposit laboratory protocols in protocols.io, where a protocol can be assigned its own identifier (DOI) such that it can be cited independently in the future. Additionally, PLOS ONE offers an option to publish peer-reviewed clinical study protocols. Read more information on sharing protocols at https://plos.org/protocols?utm_medium=editorial-email&utm_source=authorletters&utm_campaign=protocols

# Author comment: Author response to Decision Letter 1

**DOI:** 10.1371/journal.pntd.0014612.r002

7 Jul 2026

Attachment Submitted filename: Rebuttal letter.docx

# Editor report: Decision Letter 1

**DOI:** 10.1371/journal.pntd.0014612.r003

**Authors:** José Gutiérrez, Kalana Maduwage

21 Jul 2026

Dear Dr Monteiro,

We are pleased to inform you that your manuscript 'Effectiveness, Safety, and Implementation Outcomes of a Decentralization Program to Deliver Antivenoms in the Western Brazilian Amazonia: the SAVING Program' has been provisionally accepted for publication in PLOS Neglected Tropical Diseases.

Before your manuscript can be formally accepted you will need to complete some formatting changes, which you will receive in a follow up email. A member of our team will be in touch with a set of requests.

Please note that your manuscript will not be scheduled for publication until you have made the required changes, so a swift response is appreciated.

IMPORTANT: The editorial review process is now complete. PLOS will only permit corrections to spelling, formatting or significant scientific errors from this point onwards. Requests for major changes, or any which affect the scientific understanding of your work, will cause delays to the publication date of your manuscript.

Should you, your institution's press office or the journal office choose to press release your paper, you will automatically be opted out of early publication. We ask that you notify us now if you or your institution is planning to press release the article. All press must be co-ordinated with PLOS.

Thank you again for supporting Open Access publishing; we are looking forward to publishing your work in PLOS Neglected Tropical Diseases.

Best regards,

Kalana Maduwage, MBBS, MPhil, PhD, FHEA, FRCP (UK), FRCP (Edin)

Academic Editor

PLOS Neglected Tropical Diseases

José María Gutiérrez

Section Editor

PLOS Neglected Tropical Diseases

Shaden Kamhawi

co-Editor-in-Chief

PLOS Neglected Tropical Diseases

orcid.org/0000-0003-4304-636XX

Paul Brindley

co-Editor-in-Chief

PLOS Neglected Tropical Diseases

orcid.org/0000-0003-1765-0002

***********************************************************

p.p1 {margin: 0.0px 0.0px 0.0px 0.0px; line-height: 16.0px; font: 14.0px Arial; color: #323333; -webkit-text-stroke: #323333}span.s1 {font-kerning: none

# Editor report: Acceptance letter

**DOI:** 10.1371/journal.pntd.0014612.r004

**Authors:** José Gutiérrez, Kalana Maduwage

Dear Dr. Monteiro,

We are delighted to inform you that your manuscript, "Effectiveness, Safety, and Implementation Outcomes of a Decentralization Program to Deliver Antivenoms in the Western Brazilian Amazonia: the SAVING Program," has been formally accepted for publication in PLOS Neglected Tropical Diseases.

We have now passed your article onto the PLOS Production Department who will complete the rest of the publication process. All authors will receive a confirmation email upon publication.

The corresponding author will soon be receiving a typeset proof for review, to ensure errors have not been introduced during production. Please review the PDF proof of your manuscript carefully, as this is the last chance to correct any scientific or type-setting errors. Please note that major changes, or those which affect the scientific understanding of the work, will likely cause delays to the publication date of your manuscript. Note: Proofs for Front Matter articles (Editorial, Viewpoint, Symposium, Review, etc...) are generated on a different schedule and may not be made available as quickly.

Soon after your final files are uploaded, the early version of your manuscript will be published online unless you opted out of this process. The date of the early version will be your article's publication date. The final article will be published to the same URL, and all versions of the paper will be accessible to readers.

For Research Articles, you will receive an invoice from PLOS for your publication fee after your manuscript has reached the completed accept phase. If you receive an email requesting payment before acceptance or for any other service, this may be a phishing scheme. Learn how to identify phishing emails and protect your accounts at https://explore.plos.org/phishing.

Thank you again for supporting open-access publishing; we are looking forward to publishing your work in PLOS Neglected Tropical Diseases.

Best regards,

Shaden Kamhawi

co-Editor-in-Chief

PLOS Neglected Tropical Diseases

Paul Brindley

co-Editor-in-Chief

PLOS Neglected Tropical Diseases
